# Gene expression profiling of whole blood cells supports a more efficient mitochondrial respiration in hypoxia-challenged gilthead sea bream (*Sparus aurata*)

**DOI:** 10.1186/s12983-017-0220-2

**Published:** 2017-07-06

**Authors:** Juan Antonio Martos-Sitcha, Azucena Bermejo-Nogales, Josep Alvar Calduch-Giner, Jaume Pérez-Sánchez

**Affiliations:** 1 Nutrigenomics and Fish Growth Endocrinology Group, Institute of Aquaculture Torre de la Sal, Consejo Superior de Investigaciones Científicas (IATS-CSIC), Ribera de Cabanes, E-12595 Castellón, Spain; 2 Present address: Endocrine Disruption and Toxicity of Contaminants, Department of Environment, INIA, Madrid, Spain

**Keywords:** Blood transcriptomics, Hypoxia, Limiting oxygen saturation, Mitochondrial activity, Oxphos, *Sparus aurata*

## Abstract

**Background:**

Acclimation to abiotic challenges, including decreases in O_2_ availability, requires physiological and anatomical phenotyping to accommodate the organism to the environmental conditions. The retention of a nucleus and functional mitochondria in mature fish red blood cells makes blood a promising tissue to analyse the transcriptome and metabolic responses of hypoxia-challenged fish in an integrative and non-invasive manner.

**Methods:**

Juvenile gilthead sea bream (*Sparus aurata*) were reared at 20–21 °C under normoxic conditions (> 85% O_2_ saturation) followed by exposure to a gradual decrease in water O_2_ concentration to 3.0 ppm (41–42% O_2_ saturation) for 24 h or 1.3 ppm (18–19% O_2_ saturation) for up to 4 h. Blood samples were collected at three different sampling points for haematological, biochemical and transcriptomic analysis.

**Results:**

Blood physiological hallmarks remained almost unaltered at 3.0 ppm, but the haematocrit and circulating levels of haemoglobin, glucose and lactate were consistently increased when fish were maintained below the limiting oxygen saturation at 1.3 ppm. These findings were concurrent with an increase in total plasma antioxidant activity and plasma cortisol levels, whereas the opposite trend was observed for growth-promoting factors, such as insulin-like growth factor I. Additionally, gene expression profiling of whole blood cells revealed changes in upstream master regulators of mitochondria (*pgcβ* and *nrf1*), antioxidant enzymes (*gpx1, gst3,* and *sod2*), outer and inner membrane translocases (*tom70, tom22, tim44, tim10,* and *tim9*), components of the mitochondrial dynamics system (*mfn2, miffb, miro1a,* and *miro2*), apoptotic factors (*aifm1*), uncoupling proteins (*ucp2*) and oxidative enzymes of fatty acid β-oxidation (*acca2, ech,* and *hadh*), the tricarboxylic acid cycle (*cs*) and the oxidative phosphorylation pathway. The overall response is an extensive reduction in gene expression of almost all respiratory chain enzyme subunits of the five complexes, although mitochondrial-encoded catalytic subunits and nuclear-encoded regulatory subunits of Complex IV were primarily increased in hypoxic fish.

**Conclusions:**

Our results demonstrate the re-adjustment of mitochondrial machinery at transcriptional level to cope with a decreased basal metabolic rate, consistent with a low risk of oxidative stress, diminished aerobic ATP production and higher O_2_-carrying capacity. Taken together, these results suggest that whole blood cells can be used as a highly informative target tissue of metabolic condition.

**Electronic supplementary material:**

The online version of this article (doi:10.1186/s12983-017-0220-2) contains supplementary material, which is available to authorized users.

## Background

Among the abiotic factors, dissolved oxygen (O_2_) is particularly important as the major limiting factor of fish aerobic metabolism [1, 2]. When regulatory mechanisms are no longer sufficient to maintain the O_2_ consumption rate (MO_2_), further reductions in MO_2_ occur at a certain level of O_2_ saturation [3]. This threshold is termed the limiting oxygen saturation (LOS) in fed fish able to maintain a routine metabolic rate, and according to the oxystatic control theory of feed intake, fish adjust their feed intake to meet dietary O_2_ demands [4]. Therefore, changes in LOS, produced by fluctuations in O_2_ solubility associated with variations in water temperature, should be considered and regulated to ensure a non-compromised physiological function and guarantee the welfare of farmed fish fed high or low O_2_-demanding diets [5, 6]. This regulation is mediated through O_2_ sensors that trigger anaerobic metabolic rates to compensate for the decreasing aerobic ATP production [7, 8]. For this purpose, eukaryotic cells switch from mitochondrial oxidative phosphorylation (OXPHOS) to the less efficient anaerobic glycolytic pathway, which induces stress and lactic acidosis (reviewed in [9]). The hallmarks of human muscle adaptation to hypoxia are a decrease in muscle oxidative capacity concomitant with a decrease in aerobic work capacity [10, 11]. In this regard, hypo-metabolic states should be considered as part of the adaptive response to hypoxia instead of a negative result in hypoxia-tolerant individuals [12] since this metabolic depression prevents the accumulation of toxic by-products from anaerobic metabolism [13].

In fish, microarray gene expression profiling of liver and skeletal muscle demonstrated that metabolic suppression is a key adaptive strategy in the hypoxic goby fish, *Gillichthys mirabilis,* to drive energy resources from growth towards metabolic processes that are essential for hypoxia survival [14]. However, in *Fundulus grandis,* both cardiac and hepatic tissues displayed increases in the gene expression of different enzyme subunits of the OXPHOS pathway in response to short-term hypoxic exposure [15]. Similarly, confounding results have been reported in European sea bass (*Dicentrarchus labrax*), as early life exposure to moderate hypoxia has long-lasting detrimental effects on growth performance with no improvement of hypoxia tolerance in juvenile fish despite the enhanced expression of glycolytic enzymes, which are target genes of hypoxia-inducible factors [16]. Whether this response is tissue- or fish species-specific remains unclear. Importantly, the red blood cells (RBC) of fish and almost all amphibians, reptiles and birds retain a nucleus and functional mitochondria [17]. These RBCs present new research opportunities, and previous research attempts have demonstrated that the expression of mitochondrial uncoupling proteins is highly regulated by hypoxia stimuli in the whole blood cells of gilthead sea bream (*Sparus aurata*) [8]. Certainly, fish microarray meta-analysis revealed that mitochondria are particularly sensitive to cellular stress triggered by a wide range of nutritional and environmental stress stimuli [18]. Hence, a PCR-array containing 88 mitochondrial-related markers has been useful to examine changes in hepatic and muscle metabolism in response to short-term fasting [19] or aquaculture stressors that mimic thermal stress and daily operational farming activities in gilthead sea bream [20]. There is little information on blood transcriptomics, and the aim of the present study was to provide new insights into the regulation and adaptive responses of hypoxic metabolism in fish, combining non-invasive transcriptional approaches based on mitochondrial markers with conventional measures of blood haematology and biochemistry. This type of approach is crucial to determine whether samples collected without sacrificing animals provide a reliable measure of mitochondrial functioning and energy metabolism at the level of the whole organism.

## Methods

### Animal care

Juvenile gilthead sea bream of Atlantic origin (Ferme Marine du Douhet, Bordeaux, France) were reared from early life stages at the indoor experimental facilities of the Institute of Aquaculture Torre de la Sal (IATS-CSIC, Castellón, Spain) under natural photoperiod and temperature conditions at our latitude (40°5′N; 0°10′E). Seawater was pumped ashore (open system) and filtered through a 10-μm filter. The O_2_ content of water effluents under standard conditions remained consistently higher than 85% saturation, and unionised ammonia under both control and experimental conditions remained below toxic levels (<0.02 mg/L). For sampling, the fish were anaesthetised using 3-aminobenzoic acid ethyl ester (100 mg/L), and blood was drawn from caudal vessels using EDTA-treated syringes.

### Experimental setup and sampling

**Fig. 1:**
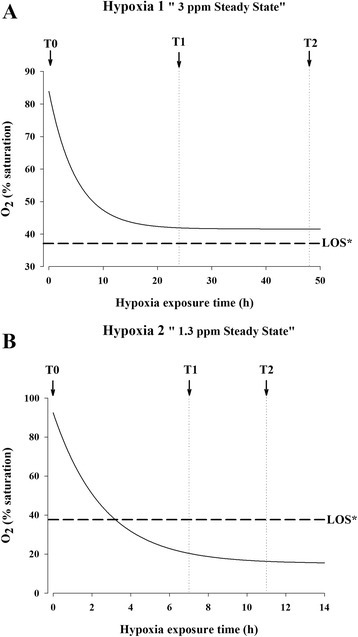
Water O_2_ kinetics in fish exposed to hypoxic conditions. The steady-state was set at (**a**) 41–42% O_2_ saturation (3 ppm) or (**b**) 18–19% O_2_ saturation (1.3 ppm). Sampling points (T0, T1 and T2) are indicated with arrowheads. LOS was calculated according to Remen et al. [5]

Juvenile fish of 230–260 g body weight were distributed in 500-L tanks (16 fish per tank) allocated in a re-circulatory system equipped with physical and biological filters and programmable temperature. The water temperature was maintained at 20–21 °C. Fish were fed daily to visual satiety using a commercial diet (INICIO Forte 824/EFICO Forte 824; BioMar, Palencia, Spain), and all fish were fasted during the hypoxia challenges. The water conditions for the control fish (normoxic fish) remained unchanged, whereas hypoxic fish experienced a gradual decrease in the water O_2_ concentration until reaching i) 3.0 ppm (41–42% O_2_ saturation; moderate hypoxia, H1) for 24 h or ii) 1.3 ppm (18–19% O_2_ saturation; severe hypoxia, H2) for up to 4 h in two different hypoxic tests (Fig. 1). Both low dissolved O_2_ levels tested were obtained by the cessation of normal aeration in the tank, achieving an accurate balance between the consumption rates of the animals and the supply of clean and oxygenated water by means of an electrovalve within the established O_2_ steady-state condition.

In each test, normoxic or hypoxia-challenged fish were sampled at three different sampling points after decreasing the water O_2_ concentration (8 fish per time and condition): i) H1: T0, T1 (24 h), T2 (48 h), and ii) H2: T0, T1 (7 h), T2 (11 h). One blood aliquot (150 μL) was directly collected into a microtube containing 500 μL of stabilising lysis solution (REAL Total RNA Spin Blood Kit, Durviz, Valencia, Spain) and stored at −80 °C until total RNA extraction. Other aliquots were processed for haematocrit (Hc) and haemoglobin (Hb) determinations. The remaining blood was centrifuged at 3000 × *g* for 20 min at 4 °C, and the plasma samples were frozen and stored at −20 °C until biochemical and hormonal analyses were performed.

### Blood biochemistry and hormonal parameters

Hc was measured using heparinised capillary tubes centrifuged at 1500 × *g* for 30 min in a Sigma 1–14 centrifuge (Sigma, Osterode am Harz, Germany). The Hb concentration was assessed using a Hemocue Hb 201+ (Hemocue, Ängelholm, Sweden). Plasma glucose was analysed using the glucose oxidase method (Thermo Electron, Louisville, CO, USA). Blood lactate was measured in deproteinised samples (perchloric acid 8%) using an enzymatic method based on the use of lactate dehydrogenase (Instruchemie, Delfzijl, The Netherlands). Total antioxidant capacity in plasma samples was measured using a commercial kit (Cayman Chemical, Ann Arbor, MI, USA) adapted to 96-well microplates. This assay relies on the ability of the antioxidants in the samples to inhibit the oxidation of ABTS (2,2′-azino-di-[3-ethylbenzothiazoline sulphonate]) to the ABTS radical cation by metamyoglobin, a derivatised form of myoglobin. The capacity of the sample to prevent ABTS oxidation was compared with that of Trolox (water-soluble tocopherol analogue) and quantified as mM Trolox equivalents. Plasma cortisol levels were analysed using an EIA kit (Kit RE52061 m IBL, International GmbH, Germany). The limit of detection of the assay was 2.46 ng/mL with intra- and inter-assay coefficients of variation lower that 3% and 5%, respectively. Plasma insulin-like growth factors (Igf) were extracted using acid-ethanol cryoprecipitation [21], and the concentration was measured using a generic fish Igf-I RIA validated for Mediterranean perciform fish [22]. The sensitivity and midrange of the assay were 0.05 and 0.7–0.8 ng/mL, respectively.

### Gene expression analysis

Total RNA from total blood cells was extracted using the REAL Total RNA Spin Blood Kit (Durviz) including a DNase step. The RNA yield was >2.5 μg, with absorbance measures (A_260/280_) of 1.9–2.1. The cDNA was synthesised using the High-Capacity cDNA Archive Kit (Applied Biosystems, Foster City, CA, USA) with random decamers and 500 ng of total RNA in a final volume of 100 μL. Reverse transcription (RT) reactions were incubated for 10 min at 25 °C and 2 h at 37 °C. Negative control reactions were run without the RT enzyme. qPCR was performed using an Eppendorf Mastercycler Ep Realplex Real-Time Detection System (Eppendorf, Wesseling-Berzdorf, Germany). Diluted RT reactions were conveniently used for qPCR assays in 25 μL volume in combination with a SYBR Green Master Mix (Bio-Rad, Hercules, CA, USA) and specific primers at a final concentration of 0.9 μM (Additional file 1: Table S1). The 96-well PCR-array layout was designed for the simultaneous profiling of a panel of 85 mitochondrial genes under uniform cycling conditions and associated with different biological processes, such as molecular chaperones (7), antioxidant defence (8), transcription factors (5), outer and inner membrane translocation (8), mitochondrial dynamics and apoptosis (10), fatty acid oxidation and the tricarboxylic acid cycle (5), OXPHOS (41) and respiration uncoupling (1). The programme used for PCR amplification included an initial denaturation step at 95 °C for 3 min, followed by 40 cycles of denaturation for 15 s at 95 °C and annealing/extension for 60 s at 60 °C. All the pipetting operations were conducted using an EpMotion 5070 Liquid Handling Robot (Eppendorf, Hamburg, Germany) to improve data reproducibility. The efficiency of PCRs (>92%) was assessed, and the specificity of the reactions was verified through an analysis of melting curves (ramping rates of 0.5 °C/10 s over a temperature range of 55–95 °C) and linearity of serial dilutions of the RT reactions (>0.99). Fluorescence data acquired during the extension phase were normalised using the delta-delta C_T_ method [23]. A range of potential housekeeping genes (*β-actin*, *cox4a*, *elongation factor 1*, *α-tubulin* and *18S rRNA*) was initially tested for gene expression stability using Genorm software. The most stable gene in relation to different experimental conditions (normoxia and hypoxia) was *cox4a* (M score = 0.31); therefore, this gene was used as the housekeeping gene in the normalisation procedure. For multi-gene analysis, the data on gene expression were in reference to the expression level of *sod1* obtained in normoxic fish, for which a value of 1 was arbitrarily assigned (Table 1).

**Table 1:**
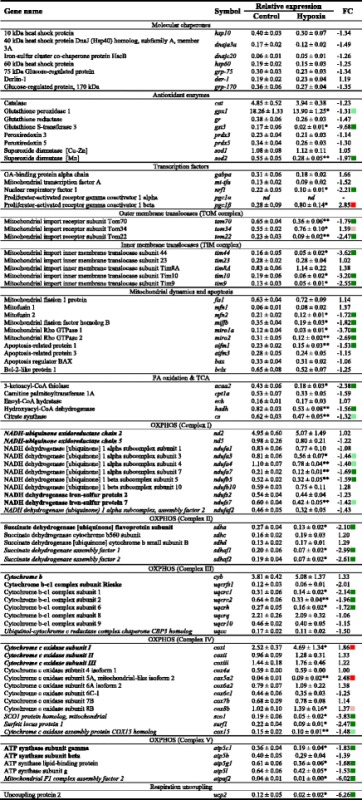
Relative gene expression of mitochondrial-related genes in total blood cells

Gilthead sea breams were exposed to normoxic (oxygen saturation > 85%) and hypoxic (1.3 ppm, oxygen saturation = 18–19%) conditions. Data are presented as the mean ± SEM (*n* = 7–8). Statistically significant differences between normoxic and hypoxic fish are indicated (*P < 0.05, **P < 0.01; Student’s *t* test). *nd*: non-detected. Gene names of mitochondrial-encoded catalytic subunits of the OXPHOS pathway are highlighted in bold and italicised. Gene names of nuclear-encoded catalytic subunits of the OXPHOS pathway are highlighted in bold. Gene names of nuclear-encoded regulatory subunits are presented in normal font. Gene names of nuclear-encoded assembly factors are italicised. Square symbols are used for up- (red) and down-regulated genes (green)

This manuscript follows the ZFIN Zebrafish Nomenclature Guidelines for gene and protein names and symbols (https://wiki.zfin.org/display/general/ZFIN+Zebrafish+Nomenclature+Guidelines).

### Statistical analysis

The data on biochemical and hormonal parameters were analysed using two-way analysis of variance (ANOVA), followed by the Holm-Sidak test. The data on gene expression were analysed using Student’s *t* test. The significance level was set at *P* < 0.05. All analyses were performed using SigmaPlot Version 13 for Windows.

## Results

### Hypoxic effects on blood haematology and biochemistry

**Fig. 2:**
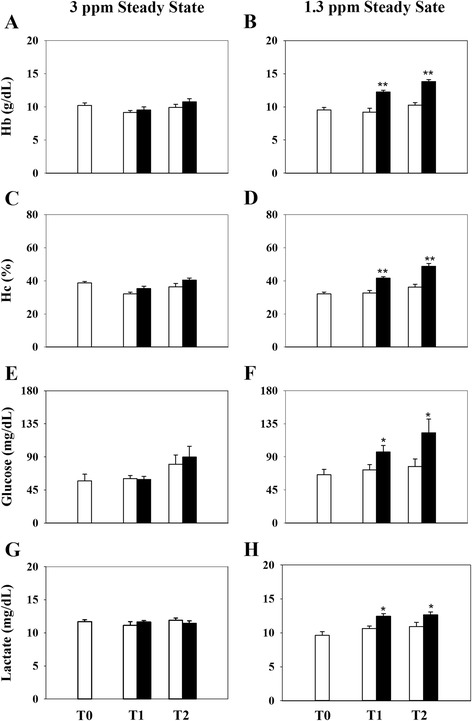
Effects of normoxia (*white bars*) and hypoxia (*black bars*) on blood haematology and biochemistry. Hypoxia levels were set above (**a**, **c**, **e**, **g**) or below (**b**, **d**, **f**, **h**) the LOS. Data are presented as the mean ± SEM (*n* = 7–8). Statistically significant differences between normoxic and hypoxic fish are indicated (*P < 0.05, **P < 0.01; two-way analysis of variance (ANOVA) followed by the Holm-Sidak test)

**Fig. 3:**
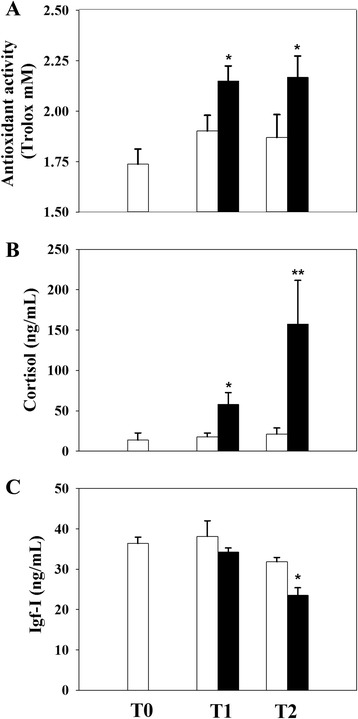
Effects of normoxia (*white bars*) and hypoxia (*black bars*) below the LOS on plasma parameters. Antioxidant activity (**a**), cortisol (**b**) and Igf-I (**c**). Data are presented as the mean ± SEM (*n* = 7–8). Statistically significant differences between normoxic and hypoxic fish are indicated (*P < 0.05, **P < 0.01; two-way analysis of variance (ANOVA) followed by the Holm-Sidak test)

Over the course of the first hypoxia test (H1, 41–42% O_2_ saturation), measurements of haematological parameters and plasma glucose and lactate levels remained unaltered in both normoxic (>85% O_2_ saturation) and hypoxia-challenged fish (Fig. 2a, c, e and g, respectively). In contrast, these parameters significantly increased in fish exposed to severe hypoxia (H2, 18–19% O_2_ saturation) (Fig. 2b, d, f and h). The same trend was observed for total plasma antioxidant activity and plasma cortisol levels (Fig. 3a, b), although the cortisol increase was more pronounced at the last sampling point. The opposite regulation was observed for circulating Igf-I, although a statistically significant effect was observed at the last sampling point (Fig. 3c). No variations in all the parameters studied were observed in fish maintained under normoxic conditions in sub-experiment H2.

### Hypoxic effects in whole blood cell gene expression profiling

Based on the results of hormonal and metabolic parameters, gene expression profiling of whole blood cells was restricted to the last sampling point of the severe hypoxia experiment (H2). The relative gene expression and fold-changes (FC) of mitochondrial-related genes are summarised in Table 1. For easier interpretation and visualisation of the results, the FC of differentially expressed genes is indicated using square symbols in red (up-regulated) or green (down-regulated). With the exception of *pgc1α,* all the genes included in the array were detected in all samples analysed. Among these genes, 41 out of 84 were differentially expressed, and the overall response involved repressed expression in response to severe hypoxia. This response was mediated by antioxidant enzymes (*gpx1*, *gst3*, and *sod2*), the transcription factor *nrf1*, outer and inner membrane translocases (*tom70*, *tom22*, *tim44*, *tim10*, and *tim9*), markers of mitochondrial dynamics and apoptosis (*mfn2*, *miffb*, *miro1a*, *miro2*, and *aifm1*), fatty acid β-oxidation (*acaa2* and *hadh),* tricarboxylic acid cycle (*cs*), respiration uncoupling (*ucp2*) and respiratory enzyme subunits of Complex I (*ndufa3*, *ndufa4*, *ndufa7*, *ndufb5*, and *ndufs7*), Complex II (*sdha*, *sdhaf1*, and *sdhaf2*), Complex III (*uqcrc1*, *uqcrc2*, and *uqcrh*) and Complex V (*atp5c1*, *atp5g1*, *atp5l*, and *atpaf2*), encoded by either mitochondrial or nuclear DNA. The nuclear-encoded assembly factors of Complex IV (*sco1*, *surf1*, and *cox15*) were also significantly down-regulated, but the opposite trend was observed for catalytic (*coxi*) and regulatory *(cox5a2* and *cox8b*) enzyme subunits of mitochondrial or nuclear origin, respectively. This up-regulation was also observed for the transcription factor *pgc1ß* and the outer membrane translocase *tom34*. The molecular chaperones were the only factors that did not significantly change under hypoxic conditions, although the overall trend was a down-regulation in hypoxic fish.

## Discussion

Studies in gilthead sea bream have indicated that the response of this species to a progressive decline in O_2_ concentration is to reduce its swimming activity, indicative of an increasing metabolic stress and/or a coping strategy to prolong survival time when hypoxia cannot be avoided [5]. In the same study, the threshold level of LOS determined in 400-g fish varied from 17% O_2_ saturation at 12 °C and 36% O_2_ saturation at 20 °C. These O_2_ concentrations can be implemented in aquaculture as a lower limit for acceptable decreases in O_2_ concentration with respect to the physiological function and welfare of farmed gilthead sea bream. Therefore, as further explained below, it is not surprising that data on blood biochemistry and haematology in fish exposed to O_2_ concentrations above the LOS did not significantly vary after 24 h of hypoxia challenge. In contrast, a consistent response, exacerbated over time, was observed for blood parameters measured a few hours after exposure to O_2_ concentrations below the LOS. In this case, the gene expression profile of whole blood cells was analysed, and the molecular signatures of hypoxic fish revealed important changes consistent with reduced but more efficient aerobic ATP production.

Living organisms are characterised by continuous switching between resting and active states, which includes long resting periods with low ATP production [24]. Similarly, the response to hypoxia has two main aspects characterised by defence and rescue, where the early defence stage is achieved by reducing energy needs (hypo-metabolic state) and the dependence on aerobic metabolism [25]. In the case of gilthead sea bream, the antioxidant defences in fish fed diets supplemented with methionine and white tea were insufficient to avoid oxidative stress under moderate hypoxia induced by 40% O_2_ saturation at 22–23 °C [26]. However, LOS increases with decreasing temperature [6], and the results of the present study showed that all measured haematological and biochemical parameters remained mostly unaltered in fish maintained at 20–21 °C and 41–42% O_2_ saturation. In contrast, a pronounced increase in Hc, Hb and plasma glucose and lactate levels was reported after exposure to severe hypoxia (18–19% O_2_ saturation) for 4 h under steady-state conditions. Indeed, this rapid response could reflect an increase in blood O_2_-carrying capacity [27] associated in the short term with erythrocyte release from a storage organ or with a reduction in plasma volume rather than the formation of new Hb [28]. Consistent with [8], this finding likely reflects metabolic changes mediated by O_2_ sensors that drive the shift of the redox cellular status of NADH to a more reduced form with a rapid recycling of NAD^+^ to NADH. Certainly, hypoxic situations must improve and adjust the metabolic and O_2_-carrying capacities of challenged fish to cope and reach internal homeostasis [29]. The trigger observed in plasma antioxidant capacity after acute and severe hypoxia demonstrates a general decrease in metabolic rates that also reflects the aerobic/anaerobic shift of metabolism [25, 30, 31].

The increase in plasma cortisol levels observed after severe hypoxia indicates a stressful scenario in the experimental model used in the present study. Other common features of hypoxic and stress conditions include a decrease in plasma Igf-I levels and concomitant growth inhibition [32, 33]. In this sense, a characteristic response in challenged gilthead sea bream produced by crowding, and presumably also through hypoxia, is the overall down-regulated expression of hepatic *igf*s and growth hormone receptors [34]. Studies in rodents support the involvement of the Gh/Igf system in the regulation of key antioxidant enzymes, ROS production and scavenging as well mitochondrial biogenesis and activity [35, 36]. However, thus far, the precise mechanisms underlying these Gh/Igf-mediated effects remain unexplored in fish. Moreover, confounding results have been reported for the aerobic/anaerobic shift during hypoxia exposure, although studies in the euryoxic mudsucker *Gillichthys mirabilis* showed a tissue-specific gene regulation resulting in suppressed protein synthesis in skeletal muscle and enhanced anaerobic ATP production in the liver tissue [14]. Similarly, zebra fish (*Danio rerio*) embryos survive during severe hypoxia (0–5% O_2_ saturation) through changes in the gene and protein expression of master regulators of O_2_ homeostasis, such as the hypoxia-inducible factor 1 alpha (*hif-*1α/Hif*-1α*) [37–40]. Additionally, long-term adaptive responses in the gene expression of several pathways related to cell architecture, cell division and energy metabolism have been underlined in the gills of adult hypoxic fish [41]. In our experimental model, this hypothesis was perfectly consistent with hypoxic-mediated effects on mitochondrial-related markers of blood cells (see below).

Most mitochondrial proteins are encoded by nuclear DNA; thus, a healthy metabolic mitochondria phenotype is highly dependent on the protein import system, which involves two assembly complexes: the translocases of the outer membrane (TOM complex) and the translocases of the inner membrane (TIM complex) (see [42, 43] for review). Thus, as demonstrated in mammalian cells [44], the TOM/TIM complex is highly inducible and regulated at both transcriptional and post-transcriptional levels under conditions of chronic stress or energy deficit to ensure the maintenance of adequate mitochondrial protein import rates. Similarly, juveniles of gilthead sea bream exhibit a clear up-regulation in the gene expression of hepatic protein subunits of the TOM/TIM complex in response to aerobic energy stimuli after exposure to cyclic decreases in water temperature [20]. Conversely, the present study demonstrated that severe hypoxia induced a pronounced down-regulation of *tom70* and *tom22* subunits in whole blood cells concurrent with decreases in mRNAs encoding protein subunits of TIM23 (*tim44*) and TIM22 (*tim10* and *tim9*) complexes. In addition, co-expression analyses revealed the up-regulation of *tom34*, which acts as a co-chaperone of the Hsp70/Hsp90 complex, inhibiting mitochondrial protein translocation when expressed in excess [45]. Taken together, these findings suggest in hypoxic fish an orchestration of the TOM/TIM complex that could enable adjustments in mitochondrial protein translocation to reduce plasma oxidative capacity and the risk of oxidative stress, a feature that is consistent with the down-regulated expression of markers of ROS production and scavenging, including *ucp2*, mitochondrial superoxide dismutase (*sod2*), enzymes of the glutathione system (*gpx1* and *gst3*) and enzymes of fatty acid β-oxidation and TCA (*acaa2*, *hadh*, and *cs*). Importantly, the same trend was observed for mitochondrial (*hsp10*, *dnaja3a*, *dnajc20*, *hsp60*, and *grp-75*) and endoplasmic reticulum (*grp-170*) molecular chaperones, suggesting that proper protein folding was primarily assured in the blood cells of gilthead sea bream under the depressed metabolism induced by hypoxia exposure. Similarly, severe hypoxia did not induce the gene expression of heat shock proteins in rainbow trout (*Oncorhynchus mykiss*) RBCs cultured in vitro when the hypoxia challenge was not accompanied by a heat shock treatment [46].

Mitochondrial dynamics is an essential process that adapts mitochondria morphology to the bioenergetics requirements of the cell (see [47] for review). The mechanism of this biological process involves the balance of two opposing procedures (fusion and fission), but it is also greatly affected by the “railways” used by the mitochondria to move inside the cells. The functionality of these organelles favours the redistribution of mitochondria within the cell to ensure high oxidative capacity under conditions of high energy demand, enabling the removal of dysfunctional or damaged mitochondria. This mechanism is highly conserved from yeast to mammals [48], and the molecular identity of major components of the fusion (*mfn1* and *mfn2*) and fission (*fis1* and *miffb*) system, as well as those of the MIRO system (*miro1a* and *miro2*) has been characterised in gilthead sea bream and uploaded to public database repositories [20]. Nevertheless, experimental evidences demonstrated that the gene expression of some of these effectors is highly induced in response to aerobic stimuli after cold-water exposure. In contrast, in the present study, severe hypoxia significantly repressed the expression of most components of this biological process (*mfn2, miffb, miro1a,* and *miro2*), including the well-known mitochondrial apoptotic factor *aifm1*. Consistently, the knockout of the transcriptional regulator *pgc1ß* is associated with a selective reduction in the expression in mice [49]. The lack of Pgc1*ß* also impaired the thermogenic response of adipose tissue and hepatic lipid metabolism in response to high fat dietary loads [50]. Therefore, Pgc1*ß* is essential for proper metabolic tuning in stress situations, contributing to the maintenance of the basal expression of mitochondrial and metabolic-related genes. However, in the present experimental model, the opposite regulation was observed for *pgc1ß* and *mfn2*, suggesting that the up-regulated expression of *pgc1ß* was more a consequence than the cause of the overall repressed expression of mitochondria-related genes. This notion was supported by the observation that the mitochondrial transcription factor *nrf1*, another target gene of *pgc1ß* [50], was also down-regulated in hypoxia-challenged fish. Notably, despite the overlapping gene expression of *pgc1ß* and its homologue *pgc1α*, the compensation of Pgc1α or Pgc1ß functions was not completely observed in Pgc1α or Pgc1ß knockout rodents [51–53]. In the case of gilthead sea bream blood cells, this effect is more exacerbated because *pgc1α* mRNAs were almost undetectable in both normoxic and hypoxic fish, although the expression of this gene at noticeable levels has previously been reported in other tissues of this fish species [20]. Whether this effect is part of the evolutionary pressure to select the conservation of functional mitochondria in the nucleated RBCs of non-mammalian vertebrates remains to be established [17].

The ultimate effector for coping with changes in energy needs and aerobic ATP production is the regulation of the OXPHOS pathway, which comprises five enzyme complexes (I-V) with catalytic enzymatic subunits encoded by both nuclear or mitochondrial DNA, whereas the enzyme subunits with regulatory or assembly properties are strictly of nuclear origin [19]. Changes in the enzymatic activities of the OXPHOS pathway have been studied for many years both in mammals and fish (e.g., [54–56]). Little is known at the molecular level, although gene expression profiling of liver, skeletal muscle and cardiac muscle tissues revealed that both the direction and magnitude of change is highly dependent on the metabolic capabilities of each tissue [8]. Thus far, the molecular fingerprinting of the OXPHOS pathway remains primarily unexplored in blood cells, and this is the first to address the specific regulation of this pathway in response to environmental stressors, evidenced by the general depletion of several components of Complexes I, II, III and V in response to severe hypoxic stimuli. Assembly factors of Complex IV (*sco1*, *surf1* and *cox15)* were also down-regulated in the present experimental model. These enzyme subunits play an important role in energy production, and mutations or defects in these molecules produce adverse effects in the appropriate function of the OXPHOS pathway in mammals [57–61]. However, this observation contrasted with the overall overexpression of catalytic and regulatory subunits of Complex IV, which was statistically significant for the catalytic *coxi* and the regulatory *cox5a2* and *cox8b* subunits. CoxI protein is encoded by mitochondrial DNA and represents one of the largest subunits of Complex IV, which contains the bimetallic centre where O_2_ binds and is reduced to H_2_O [62, 63]. In addition, the observed increase in the gene expression of Cox5a and Cox8 family subunits highlights their importance during the completion of the holocomplex monomer, which contains the functional structure of the cytochrome *c* binding site (see [64] for review). Therefore, we hypothesised that the net effect should be a reduced mitochondrial ATP production due to the overall suppression of mRNAs encoding the enzyme subunits of Complexes I, II, III and IV, although the opposite regulation of the catalytic/regulatory components of Complex IV should be accompanied by subsequent mechanisms that allow a better exploitation of available oxygen in the most energetically favorable way. Modifications in mitochondrial properties also occur in other vertebrates, and the hypo-metabolic steady-state observed in overwintering frogs (*Rana temporaria*) occurred during hypoxic submergence by increases in mitochondrial O_2_ affinity and a reduction in resting (state 4) and active (state 3) respiration rates in mitochondria isolated from skeletal muscle [65]. Similarly, early studies in the freshwater European eel (*Anguilla anguilla*) suggest that the efficiency of OXPHOS is increased after acclimation to high hydrostatic pressure, decreasing the enzymatic activity of Complex II in red muscle, whereas that of Complex IV is significantly increased [66]. This situation would enable a reduction in the electron leak and the optimisation of the respiratory chain. Similarly, more recent studies in gilthead sea bream have revealed that the gene expression ratio of the enzyme subunits of Complexes I and IV is altered in heart and liver tissue during the recovery state after severe hypoxia exposure [67]. Thus, as reviewed by [68], it is now evident that variations in the mitochondrial efficiency of ATP production exist among individuals, populations and environments, and even within the same individual over time. This spatial and temporal variability in mitochondrial machinery adds an additional layer of complexity to the regulation of energy metabolism, and the maintenance of aerobic metabolism is becoming recognised as a primary hypoxia survival strategy in most organisms, including fish [69]. Even so, the usage of transcriptomic analysis with other experimental approaches related to mitochondrial activity and respiration would be necessary for the better understanding about the proposed re-adjustment of mitochondrial function in hypoxia-challenged fish.

## Conclusions and future perspectives

**Fig. 4:**
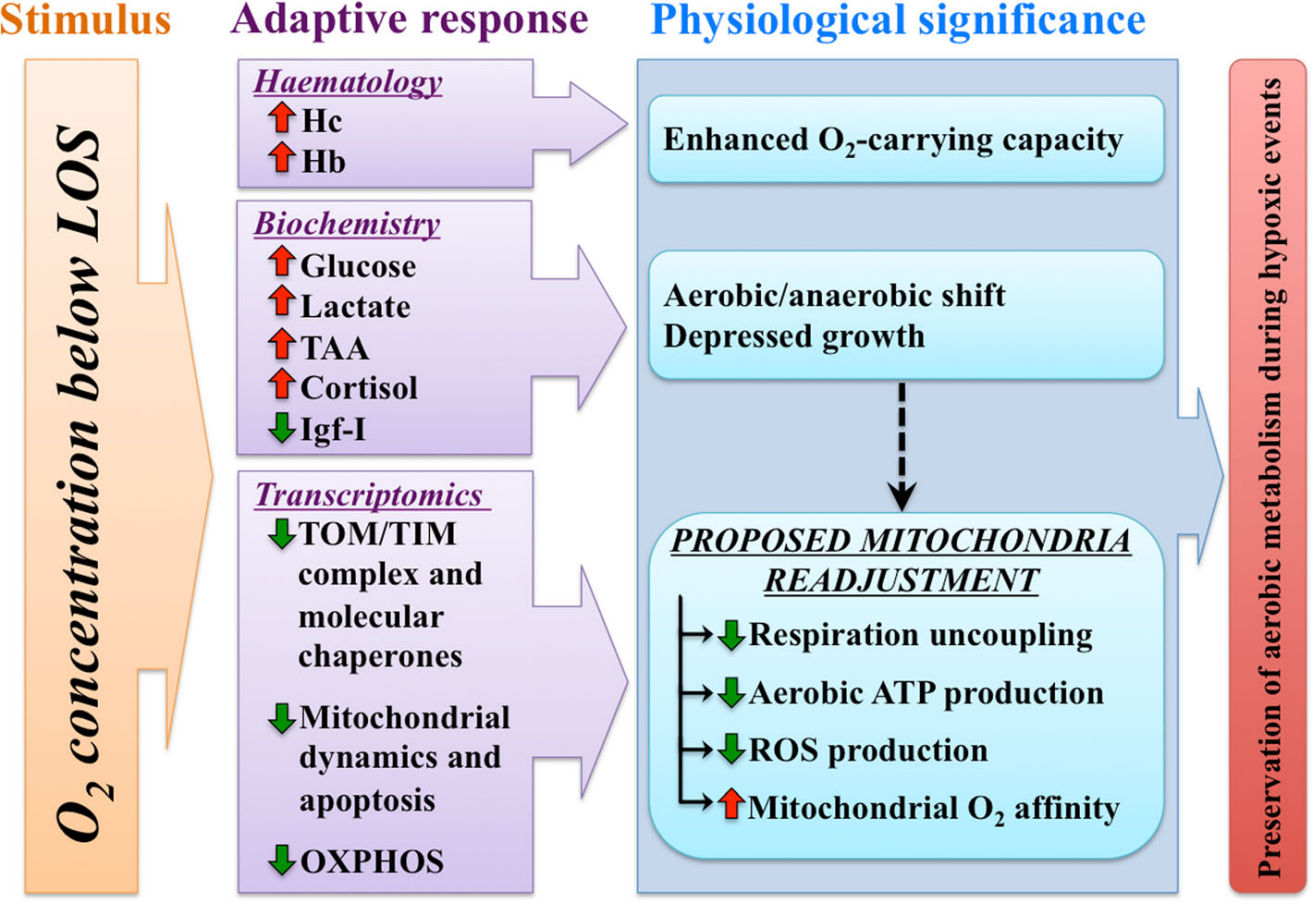
Schematic representation of the proposed model for integrative physiological responses of gilthead sea bream exposed to acute and severe hypoxia

As summarized in Fig. 4, the integrated data on blood haematology, biochemistry and transcriptomics in response to water O_2_ concentrations below the LOS highlighted an enhanced O_2_-carrying capacity as a result of higher Hc and Hb concentrations in response to strong hypoxic stimuli. Changes in plasma antioxidant capacity, as well as hormone and metabolite levels supported reduced energy needs and also reflected an aerobic/anaerobic shift. These results were further confirmed by gene expression profiling of a wide representation of mitochondrial-related markers, including antioxidant enzymes and molecular chaperones, effectors of mitochondrial dynamics and apoptosis, and key components of the respiratory chain, suggesting that the mitochondrial bioenergetics of fish blood cells are finely adjusted at the transcriptional level through changes in water O_2_ concentrations. The induced gene expression profiles of catalytic and regulatory enzyme subunits of Complex IV should be considered an adaptive process to ensure reduced but more efficient aerobic ATP production consistent with reduced respiration uncoupling, as suggested by the decreased expression of *ucp2*. These results indicate that the gilthead sea bream is a highly euryoxic fish. Further studies are underway to determine the resilience of gilthead sea bream to high rearing densities and low O_2_ concentrations, exploring the potential benefits of hypoxic preconditioning for improving the aerobic scope and swimming metabolic activity of farmed fish.

## Additional file

Additional file 1: Table S1. Forward (F) and reverse (R) primers used for real-time PCR. (DOCX 50 kb)
