# Supplementary material for: Gene expression profiling of whole blood cells supports a more efficient mitochondrial respiration in hypoxia-challenged gilthead sea bream (Sparus aurata)
Source: Front Zool. 2017 Jul 6;14:34. doi: 10.1186/s12983-017-0220-2 (PMC5501551; doi:10.1186/s12983-017-0220-2)
Supplement: Additional file 1: Table S1. — Forward (F) and reverse (R) primers used for real-time PCR. (DOCX 50 kb) [file 12983_2017_220_MOESM1_ESM.docx]

**Additional file 1: Table S1.** Forward (F) and reverse (R) primers used for real-time PCR.

| **Category** | **Gene name** | **Symbol** | **Acc. No.** |  | **Primer sequences (5’ 🡪 3’)** |
| --- | --- | --- | --- | --- | --- |
| **Molecular chaperones** | 10 kDa heat shock protein | *hsp10* | JX975224 | F | CAT GCT GCC AGA GAA GTC TCA AGG |
|  |  |  |  | R | AGG TCC CAC TGC CAC TAC TGT |
|  | 40 kDa heat shock protein DnaJ (Hsp40) homolog, subfamily A, member 3A | *dnaja3a* | JX975225 | F | CCA AAT GCT GTC TCC TCA CTG TCC TTT C |
|  |  |  |  | R | ACC TGA TAG AAG TCC TGC TTG CTG CTA |
|  | Iron-sulfur cluster co-chaperone protein HscB | *dnajc20* | JX975226 | F | GCC AGA AGC AGC CAA TAG GAT |
|  |  |  |  | R | CTT TGA GCA GGG CAG CGT CTA |
|  | 60 kDa heat shock protein | *hsp60* | JX975227 | F | TGT GGC TGA GGA TGT GGA TGG AGA G |
|  |  |  |  | R | GCC TGT TGA GAA CCA AGG TGC TGA G |
|  | 75 kDa Glucose-regulated protein | *grp-75* | DQ524993 | F | TCC GGT GTG GAT CTG ACC AAA GAC |
|  |  |  |  | R | TGT TTA GGC CCA GAA GCA TCC ATG |
|  | Derlin-1 | *der-1* | JQ308825 | F | ACT GCC TCG GTT GCC TTT CC |
|  |  |  |  | R | TGG CTG TCA CAA GTC TCC AGA TAT G |
|  | Glucose-regulated protein, 170 kDa | *grp-170* | JQ308821 | F | CAG AGG AGG CAG ACA GCA AGA C |
|  |  |  |  | R | TTC TCA GAC TCA GCA TTT CCA GAT TTC |
| **Antioxidant enzymes** | Catalase | *cat* | JQ308823 | F | TGG TCG AGA ACT TGA AGG CTG TC |
|  |  |  |  | R | AGG ACG CAG AAA TGG CAG AGG |
|  | Glutathione peroxidase 1 | *gpx1* | DQ524992 | F | GAA GGT GGA TGT GAA TGG AAA AGA TG |
|  |  |  |  | R | CTG ACG GGA CTC CAA ATG ATG G |
|  | Glutathione reductase | *gr* | AJ937873 | F | TGT TCA GCC ACC CAC CCA TCG G |
|  |  |  |  | R | GCG TGA TAC ATC GGA GTG AAT GAA GTC TTG |
|  | Glutathione S-transferase 3 | *gst3* | JQ308828 | F | CCA GAT GAT CAG TAC GTG AAG ACC GTC |
|  |  |  |  | R | CTG CTG ATG TGA GGA ATG TAC CGT AAC |
|  | Peroxiredoxin 3 | *prdx3* | GQ252681 | F | ATC AAC ACC CCA CGC AAG ACT G |
|  |  |  |  | R | ACC GTT TGG ATC AAT GAG GAA CAG ACC |
|  | Peroxiredoxin 5 | *prdx5* | GQ252683 | F | GAG CAC GGA ACA GAT GGC AAG G |
|  |  |  |  | R | TCC ACA TTG ATC TTC TTC ACG ACT CC |
|  | Superoxide dismutase [Cu-Zn] | *sod1* | JQ308832 | F | CCT GAC CTG ACC TAC GAC TAT GG |
|  |  |  |  | R | AGT GCC TCC TGA TAT TTC TCC TCT G |
|  | Superoxide dismutase [Mn] | *sod2* | JQ308833 | F | CCT GAC CTG ACC TAC GAC TAT GG |
|  |  |  |  | R | AGT GCC TCC TGA TAT TTC TCC TCT G |
| **Transcription factors** | GA-binding protein alpha chain | *gabpa* | JX975261 | F | CAT TGC CGT GGA CCG ATC TG |
|  |  |  |  | R | GCG TGT GAC CTG CTC TGA C |
|  | Mitochondrial transcription factor A | *mt-tfa* | JX975262 | F | GAG CCC GCA ACA GAA ACA GCC ATT |
|  |  |  |  | R | ACT GCT CCC TGT CCC GCT GAT AG |
|  | Nuclear respiratory factor 1 | *nrf1* | JX975263 | F | CAG ATA GTC CTG GCA GAG A |
|  |  |  |  | R | GAC CTG TGG CAT CTT GAA |
|  | Proliferator-activated receptor gamma coactivator 1 alpha | *pgc1α* | JX975264 | F | CGT GGG ACA GGT GTA ACC AGG ACT C |
|  |  |  |  | R | ACC AAC CAA GGC AGC ACA CTC TAA TTC T |
|  | Proliferator-activated receptor gamma coactivator 1 beta | *pgc1β* | JX975265 | F | TCA GAG GAA GAG GCG GAT |
|  |  |  |  | R | GAC ACA GGT GGA GGA TGG |

| **Outer membrane translocation**  **(*TOM complex*)** | Mitochondrial import receptor subunit Tom70 | *tom70* | JX975234 | F | GAG TCA GGT GGT CGA TAC A |
| --- | --- | --- | --- | --- | --- |
|  |  |  |  | R | CCA ATG AGC AGG TAG AAT GTG |
|  | Mitochondrial import receptor subunit Tom34 | *tom34* | JX975235 | F | GCT ACC GCC ACT TCT CCA CAA |
|  |  |  |  | R | TCT GTT TGG TGC CGT TCT GCT |
|  | Mitochondrial import receptor subunit Tom22 | *tom22* | JX975236 | F | CGC TCT GGG TGG GTA CTA CCT CCT T |
|  |  |  |  | R | CGA ACA CAA CAG GCA GCA CCA GGA T |
| **Inner membrane translocation**  **(*TIM complex*)** | Mitochondrial import inner membrane translocase subunit 44 | *tim44* | JX975239 | F | GAT GAC CTG GGA CAC ACT GG |
|  |  |  |  | R | TCA CTC CTC TTC CTG AGT CTG G |
|  | Mitochondrial import inner membrane translocase subunit 23 | *tim23* | JX975240 | F | CAA GTC AGG AAG TGG CGT AA |
|  |  |  |  | R | AGA GCG TAG GCA CCA GAT A |
|  | Mitochondrial import inner membrane translocase subunit Tim8A | *tim8A* | JX975245 | F | CGA CAC CAC CCT GAC CAT CAC |
|  |  |  |  | R | CGC CCT TCT GCA CCA TCT GT |
|  | Mitochondrial import inner membrane translocase subunit Tim10 | *tim10* | JX975247 | F | TAC CGC CAC ATT ACA AGG AGC |
|  |  |  |  | R | ATC CAG GCA CAC CGA CTC |
|  | Mitochondrial import inner membrane translocase subunit Tim9 | *tim9* | JX975248 | F | CGT CAA AGA TTT CAC CAC CAG AGA G |
|  |  |  |  | R | GGA GAC ACG ACT CGG AGC A |
| **Mitochondrial dynamics and apoptosis** | Mitochondrial fission 1 protein | *fis1* | JX975249 | F | TCT CAG GAA CGA GCC AGG GAA CA |
|  |  |  |  | R | CCT TGT CGA TGA GTT TCT CCA GGT CCA G |
|  | Mitofusin 1 | *mfn1* | JX975250 | F | CAT CGT TGG AGG AGT GGT GTA |
|  |  |  |  | R | CCG TAC AGT GAG GCT GAG AG |
|  | Mitofusin 2 | *mfn2* | JX975251 | F | GGG ATG CCT CAG CCT CAG AAC CT |
|  |  |  |  | R | CTG CCT GCG GAC CTC TTC CAT GTA TT |
|  | Mitochondrial fission factor homolog B | *miffb* | JX975252 | F | CGC AGC AGC ATT CCC TTC |
|  |  |  |  | R | CTC GTA CTG GAT TCG GTT CAT CT |
|  | Mitochondrial Rho GTPase 1 | *miro1a* | JX975253 | F | CAG GAC TTC TGC CGT AAG C |
|  |  |  |  | R | TAA GTG CAT CGG TCG TGT TG |
|  | Mitochondrial Rho GTPase 2 | *miro2* | JX975254 | F | TGA GGT GGA TGT GGA GGT GGA GTT |
|  |  |  |  | R | CAA GCA ACA TCA CAG GAG GCG TCT |
|  | Apoptosis-related protein 1 | *aifm1* | JX975255 | F | ACA GAG GAG TCA GGA ACC |
|  |  |  |  | R | GGA GCA GGC AAT GAA GAG |
|  | Apoptosis-related protein 3 | *aifm3* | JX975256 | F | GCA GCG GTA CAG TCT TGA ATG G |
|  |  |  |  | R | CCA GCG GAC GAG GAG CAA |
|  | Apoptosis regulator BAX | *bax* | JX975257 | F | GTG GCA GAC GGT GGG TGT TT |
|  |  |  |  | R | GCG AAT GAC GAG AAC AGT GGT GAG |
|  | Bcl-2-like protein 1 | *bclx* | JX975259 | F | CGA CAT CAC TCC TGA CAC AGC CTA C |
|  |  |  |  | R | CCG TCC TTG AAC ACC TCG TCC ATC |
| **FA oxidation**  **& TCA** | 3-ketoacyl-CoA thiolase | *acaa2* | JX975228 | F | CAT CAC TGC CCA CCT GGT TCA T |
|  |  |  |  | R | CCA ACA GCG TAC TTG CCT CCT |
|  | Carnitine palmitoyltransferase 1A | *cpt1a* | JQ308822 | F | GTG CCT TCG TTC GTT CCA TGA TC |
|  |  |  |  | R | TGA TGC TTA TCT GCT GCC TGT TTG |
|  | Enoyl-CoA hydratase | *ech* | JQ308826 | F | GCC CAA GAA GCC AAG CAA TCA G |
|  |  |  |  | R | CTT TAG CCA TAG CAG AGA CCA GTT TG |
|  | Hydroxyacyl-CoA dehydrogenase | *hadh* | JQ308829 | F | GAA CCT CAG CAA CAA GCC AAG AG |
|  |  |  |  | R | CTA AGA GGC GGT TGA CAA TGA ATC C |
|  | Citrate synthase | *cs* | JX975229 | F | TCC AGG AGG TGA CGA GCC |
|  |  |  |  | R | GTG ACC AGC AGC CAG AAG AG |

| **OXPHOS (*Complex I*)** | NADH-ubiquinone oxidoreductase chain 2 | *nd2* | KC217558 | F | TAG GTT GAA TGA CCA TCG TA |
| --- | --- | --- | --- | --- | --- |
|  |  |  |  | R | GGC TAA GGA GTT GAG GTT |
|  | NADH-ubiquinone oxidoreductase chain 5 | *nd5* | KC217559 | F | CCT AAA CGC CTG AGC CCT GG |
|  |  |  |  | R | GCT GTA AAC GAG GTG GCT AGA AGG |
|  | NADH dehydrogenase [ubiquinone] 1 alpha subcomplex subunit 1 | *ndufa1* | KC217562 | F | CGG GTT CCG TGG CAG TGG TA |
|  |  |  |  | R | TCC TGT TCC TGA TAC TCG CTT GTC TCT |
|  | NADH dehydrogenase [ubiquinone] 1 alpha subcomplex subunit 3 | *ndufa3* | KC217564 | F | TCG GAG CGT TCC TGA AGA ATG C |
|  |  |  |  | R | GAA GAG CCA TAC CTA TCA GTC CAA TAC CA |
|  | NADH dehydrogenase [ubiquinone] 1 alpha subcomplex subunit 4 | *ndufa4* | KC217565 | F | GCT CGT CTG GGC TTG AGA AAC C |
|  |  |  |  | R | GCT CTG GGT TGT TCT TGC GAT CC |
|  | NADH dehydrogenase [ubiquinone] 1 alpha subcomplex subunit 7 | *ndufa7* | KC217569 | F | CCG AGC CAC AAG TAT GCC AGC AAC TA |
|  |  |  |  | R | AGC CTC CCT GCG TCC ATC TCT G |
|  | NADH dehydrogenase [ubiquinone] 1 beta subcomplex subunit 5 | *ndufb5* | KC217580 | F | TGC GTC GGC AGA TGA GGA T |
|  |  |  |  | R | CTT GTT GAG GGT GTT CAC CTG GAA |
|  | NADH dehydrogenase [ubiquinone] 1 beta subcomplex subunit 10 | *ndufb10* | KC217585 | F | ACT GTG CCC ACG AAC TGA A |
|  |  |  |  | R | ATG CTC CCA GGT CTC CAT AG |
|  | NADH dehydrogenase iron-sulfur protein 2 | *ndufs2* | KC217589 | F | GTA TCA GAC GGC TCC AGC AGA C |
|  |  |  |  | R | AGA CCA GCC AAG TGA GCG AAT |
|  | NADH dehydrogenase iron-sulfur protein 7 | *ndufs7* | KC217594 | F | AAC GGA GGA GGC TAC TAC CAC TAC T |
|  |  |  |  | R | CGG TAC GAT TCG GTC ACA ACC TCT AAC |
|  | NADH dehydrogenase (ubiquinone) 1 alpha subcomplex, assembly factor 2 | *ndufaf2* | KC217598 | F | AGG CAG CAT ACC GAT AGA G |
|  |  |  |  | R | ACT CAT TCT TCA GCA ACT CCT |
| **OXPHOS (*Complex II*)** | Succinate dehydrogenase [ubiquinone] flavoprotein subunit | *sdha* | KC217615 | F | CAA TCT CTG GAT GAG CAG GAC TGT |
|  |  |  |  | R | GTA GGA GCG GAT GGC AGG AG |
|  | Succinate dehydrogenase cytochrome b560 subunit | *sdhc* | KC217617 | F | AGT GAC ACA CAG AGG AAC TGG AGT |
|  |  |  |  | R | CAG GGC AAA GGC GGA GAT AGC |
|  | Succinate dehydrogenase [ubiquinone] cytochrome b small subunit B | *sdhd* | KC217618 | F | CAT CAG GAG CAG CCG TAC A |
|  |  |  |  | R | CCA GAG GCA GCG TAC AGA G |
|  | Succinate dehydrogenase assembly factor 1 | *sdhaf1* | KC217619 | F | CCA GGA CAA ACC AGG CTT CAT C |
|  |  |  |  | R | GTC TTC TTG ATG CGA GCG TTC T |
|  | Succinate dehydrogenase assembly factor 2 | *sdhaf2* | KC217620 | F | CCA GCG AGT CCA TTG ACA TCA |
|  |  |  |  | R | CCC TCT TTC GGC TCT CAT ACA G |
| **OXPHOS (*Complex III*)** | Cytochrome b | *cyb* | DQ198005 | F | TGA CAG GGC TAT TCC TCG CTA TGC |
|  |  |  |  | R | AGA TGT GGG CTA CGG AAG AGA AGG |
|  | Cytochrome b-c1 complex subunit Rieske | *uqcrfs1* | KC217622 | F | GCG ACT TCG GTG GTT ACT ACT G |
|  |  |  |  | R | GCG ACC TGA GGC GTC ATA ATG |
|  | Cytochrome b-c1 complex subunit 1 | *uqcrc1* | KC217623 | F | TGT CCT GCT GTG GTT GCT GTT |
|  |  |  |  | R | CGC ACT CTG TTG TAG TCG GGT AG |
|  | Cytochrome b-c1 complex subunit 2 | *uqcrc2* | KC217624 | F | GAG CAA TTC CTC AAC ATT CG |
|  |  |  |  | R | TCT CAC CTC CAC GAT ACT G |
|  | Cytochrome b-c1 complex subunit 6 | *uqcrh* | KC217625 | F | AAG TGT GAG CAG ACT GAA C |
|  |  |  |  | R | TCA GTG TGG GAT CTG GAG |
|  | Cytochrome b-c1 complex subunit 8 | *uqcrq* | KC217627 | F | GTC AGA CAT ATT ATC ACC TA |
|  |  |  |  | R | GAT TCC CTT TGA GAA GTA |
|  | Cytochrome b-c1 complex subunit 9 | *uqcr10* | KC217628 | F | GCT GGC GAA GTC CGT CTA CAA |
|  |  |  |  | R | AGA GAA CCG CTC CAA CCA TGA TG |
|  | Ubiquinol-cytochrome c reductase complex chaperone CBP3 homolog | *uqcc* | KC217631 | F | CAG TCC TTC GTC AGA ACC GCT TT |
|  |  |  |  | R | GCA GGC TTC TTG ATC TAA CAC CTT TCC |
| **OXPHOS (*Complex IV*)** | Cytochrome c oxidase subunit I | *coxi* | KC217652 | F | GTC CTA CTT CTT CTG TCC CTT CCT GTT CT |
|  |  |  |  | R | AGG TTT CGG TCT GTA AGG AGC ATT GTA ATC |
|  | Cytochrome c oxidase subunit II | *coxii* | KC217653 | F | ACT GCC TAC ACA GGA CCT TGC C |
|  |  |  |  | R | GTC TGC TTC CAG GAG ACG GAA TTG T |
|  | Cytochrome c oxidase subunit II | *coxiii* | KC217654 | F | CCA AGC ACA CGC ATA CCA CAT A |
|  |  |  |  | R | GCG GCA ACT GCA CCT GTA |
|  | Cytochrome c oxidase subunit 4 isoform 1 | *cox4a* | JQ308835 | F | ACC CTG AGT CCA GAG CAG AAG TCC |
|  |  |  |  | R | AGC CAG TGA AGC CGA TGA GAA AGA AC |
|  | Cytochrome c oxidase subunit 5A, mitochondrial-like isoform 2 | *cox5a2* | KC217635 | F | CGC CAT CCG CAT CCT TGA |
|  |  |  |  | R | GGC TTC AAC TCT TGG ATC AGG TAG G |
|  | Cytochrome c oxidase subunit 6A isoform 2 | *cox6a2* | KC217639 | F | TGT TGG CTG CTG CGT CAC ATT C |
|  |  |  |  | R | CAG AAT CTT CCA GGT CCT CGC TCC |
|  | Cytochrome c oxidase subunit 6C-1 | *cox6c1* | KC217642 | F | TCT CTC TGT CAC TCC TGG CTG CGA TAG |
|  |  |  |  | R | CCT GGG CTC TGT CAC TGC GTA CTT G |
|  | Cytochrome c oxidase subunit 7B | *cox7b* | KC217645 | F | TCT TCT GTG TGG CTG TGT GGT CAT ACG |
|  |  |  |  | R | TTC CCA ACA GGT GAC AAA TTC CAG GTG AT |
|  | Cytochrome c oxidase subunit 8B | *cox8b* | KC217648 | F | TCC GCT GGT CCC TGT GGC TAA |
|  |  |  |  | R | CCT CCA CTG ATA TTG TGT TTG GCA GGT TTG |
|  | SCO1 protein homolog, mitochondrial | *sco1* | KC217649 | F | ACA ACA ACA AGC CCA CCA AGA |
|  |  |  |  | R | GAC AGT GAG TGA ACC CGA AGT AGA T |
|  | Surfeit locus protein 1 | *surf1* | KC217650 | F | AGA TGG AAG GTG AAG TGG AGG TGG TC |
|  |  |  |  | R | GCG TTG CTC TGT CTG CCG AAC T |
|  | Cytochrome c oxidase assembly protein COX15 homolog | *cox15* | KC217651 | F | CAT ACT AGG TCG CTG GTT AG |
|  |  |  |  | R | GAT TCC GTG AGC CTT GTG |
| **OXPHOS (*Complex V*)** | ATP synthase subunit gamma | *atp5c1* | KC217603 | F | GAG GAG AAG GTC GCC AAG CAT |
|  |  |  |  | R | GCC ACA GAG ACC ACG ATC AGA G |
|  | ATP synthase subunit beta | *atp5b* | KC217602 | F | GTA GCA CTG GTG TAT GGT CAG ATG AAC GA |
|  |  |  |  | R | TCT GGC ACG GGC ACC TGG |
|  | ATP synthase lipid-binding protein | *atp5g1* | KC217635 | F | GGA GTC GCT GGA TCT GGA GCT GG TAT T |
|  |  |  |  | R | ATA GCC AAT GAT GAG ACT GCC GAA CAC TGT |
|  | ATP synthase subunit g | *atp5l* | KC217607 | F | ACC TTC ACC TGC CGA GAT |
|  |  |  |  | R | CAG CCT GGA AGC TCT TGA TG |
|  | Mitochondrial F1 complex assembly factor 2 | *atpaf2* | KC217614 | F | CGT TCT GGC GAT GGC GAT GAT TGA CA |
|  |  |  |  | R | TGG AGA GCA GCA CAG CCT GTT CTA CG |
| **Respiration uncoupling** | Uncoupling protein 2 | *ucp2* | JQ859959 | F | CGG CGG CGT CCT CAG TTG |
|  |  |  |  | R | AAG CAA GTG GTC CCT CTT TGG TCA T |
